# Primary abdominal cocoon syndrome manifesting with Chilaiditi syndrome and intestinal obstruction: A case report

**DOI:** 10.1002/ccr3.8363

**Published:** 2023-12-27

**Authors:** Ali Tajaddini, Mohammadmehdi Fallahi, Hoda Haghshenas, Soheila‐sadat Nourmohammadi, Leila Ghahramani, Reza Shahriarirad

**Affiliations:** ^1^ Department of Surgery Shiraz University of Medical Sciences Shiraz Iran; ^2^ Student Research Committee Jahrom University of Medical Sciences Jahrom Iran; ^3^ Student Research Committee Shiraz University of Medical Sciences Shiraz Iran; ^4^ Colorectal Research Center Shiraz University of Medical Sciences Shiraz Iran; ^5^ School of Medicine Shiraz University of Medical Sciences Shiraz Iran; ^6^ Thoracic and Vascular Surgery Research Center Shiraz University of Medical Sciences Shiraz Iran

**Keywords:** abdominal cocoon syndrome, Chilaiditi syndrome, encapsulating peritoneal sclerosis, intestinal obstruction, peritoneal sclerosis

## Abstract

**Key Clinical Message:**

Abdominal cocoon syndrome and Chilaiditi syndrome are rare etiologies of bowel obstruction which have to be considered in patients with obstructive symptoms. Patients can profit from surgical management rather than non‐surgical approach.

**Abstract:**

Encapsulating peritoneal sclerosis or abdominal cocoon syndrome (ACS) is an uncommon cause of intestinal obstructions associated with encapsulation of the small bowel by a fibro collagenous sac. Clinical presentations of ACS are unspecific and most patients are diagnosed intraoperatively. Moreover, Chilaiditi syndrome is another rare cause of bowel obstruction defined by interposition of colon and liver. There is no reported relation between these two conditions and surgical intervention is the suggested approach for severe bowel obstruction following them individually. We present a case with both conditions and describe our approach. A 47‐year‐old male presented with complaints of colic abdominal pain and distention, nausea and several attacks of bilious and nonbilious vomiting, anorexia, and constipation in the last 10 days before his admission. Laboratory data were normal and abdominal X‐ray showed large dilation at the distal part of the bowel without air fluid level. The patient underwent explorative laparotomy and a mass‐like lesion containing necrotic bowel and a whitish spleen accompanied by a complete anterior‐rotated liver was found. The encapsulated bowel and the spleen were resected followed by the complete resolution of symptoms in the patient. The intestinal obstruction caused by ACS is mostly approached by surgery to prevent the fatal sequela of this condition.

## Graphical abstract

The resected part of small intestine encapsulated in a fibrous membrane, accompanied by a white lesion on the spleen.

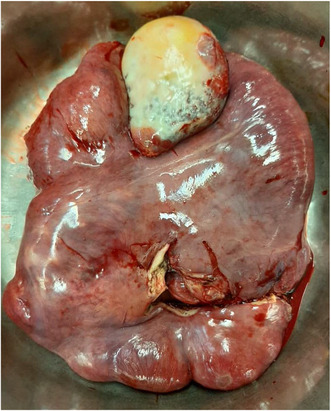

## 1 INTRODUCTION

Abdominal cocoon syndrome (ACS) or encapsulating peritoneal sclerosis (EPS) is a rare but life‐threatening condition associated with encapsulation of the small bowel by fibro collagenous cocoon‐like sac.^1^ This syndrome is more common in tropical and subtropical countries and is predominant in the male population.^2^ The clinical manifestations are not specific and preoperative diagnosis is difficult; therefore, nearly 70% of the patients are diagnosed intraoperatively.^3^ EPS can be idiopathic or secondary to a wide range of underlying causes. There are some theories that the idiopathic form related to congenital anomalies includes greater omentum absence or dysplasia and mesenteric malposition or malrotation.^4^, ^5^ Furthermore, the secondary form is associated with irritation or inflammation of the peritoneum characterized by acquired causes.^6^ Therefore, multiple congenital and acquired factors can affect the development of EPS.

Chilaiditi syndrome also is an unusual condition characterized by colon interposition and many factors including congenital malposition, functional disease, and intra‐abdominal procedures may contribute in development of this syndrome.^7^ Both ACS and Chilaiditi syndrome are rare causes of obstruction with unknown etiology.^2^, ^8^ Since ACS has a mortality rate of 50% and clinical diagnosis is mostly delayed, having high clinical suspicion and awareness of disease manifestations is needed for improving outcomes.^9^ Herein we report the clinical course of an uncommon idiopathic ACS associated with Chilaiditi syndrome presented with obstruction.

## 2 CASE PRESENTATION

A 47‐year‐old male presented to the emergency general surgery department of our referral hospital with complaints of abdominal distention, constant vomiting, and constipation that last for 10 days and did not get better by conservative therapy in outpatient setting. He was a middle‐aged man with poor general condition. His vital signs were stable. He had a history of intermittent abdominal pain and constipation, which was managed nonsurgically. His condition was accompanied by weight loss (>15% during 3 months). He had minor thalassemia, gastroesophageal reflux disease (GERD) and chronic constipation, and he was addicted to opium (3 times a day) and heavy smoking (1 pack per day). The patient had no history of previous hospitalization or surgical operation.

**FIGURE 1:**
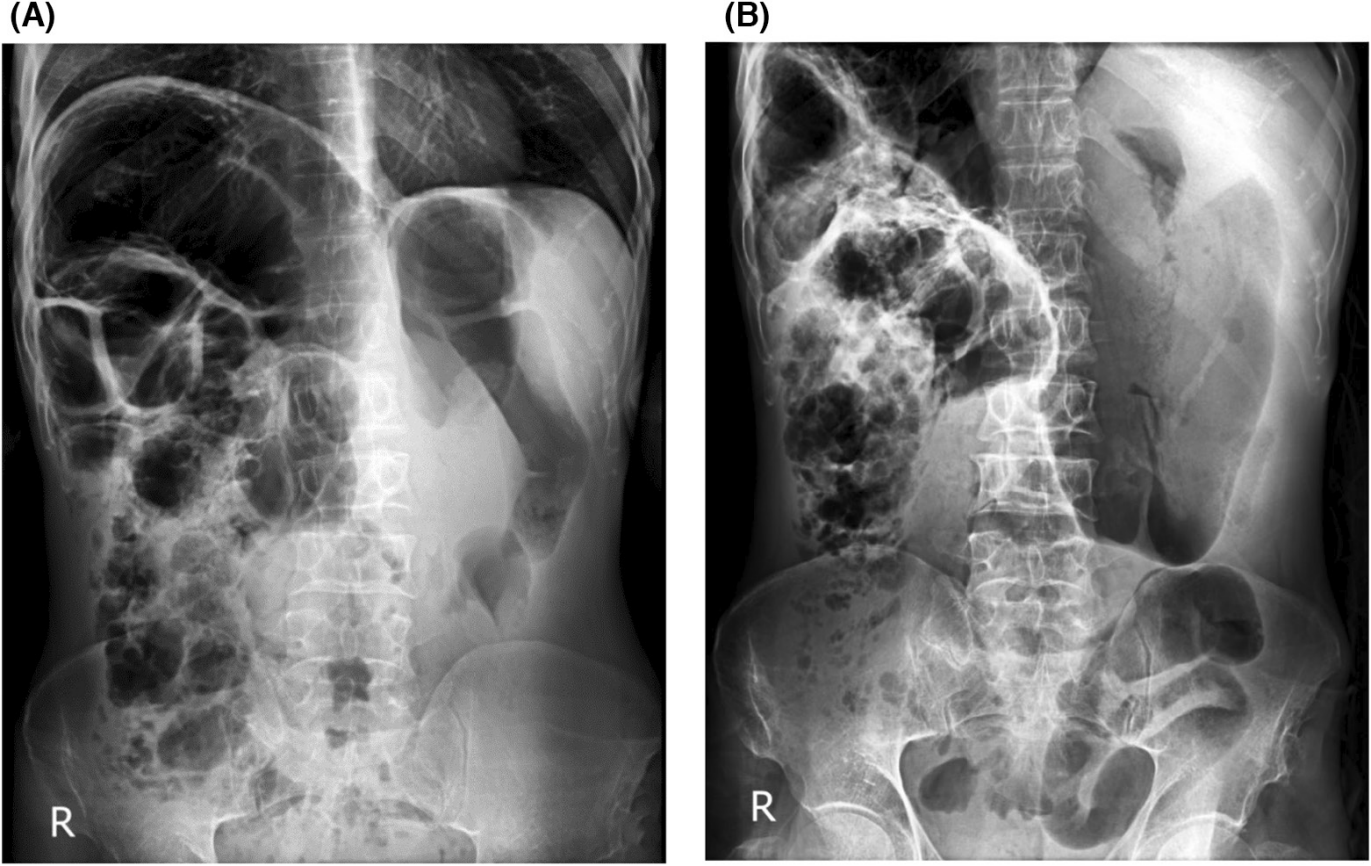
Abdominal X‐ray of the patient in (A) upright and (B) supine position, demonstrating a large dilation in the distal part of the bowel without air fluid level, with the liver not visualized in its anatomical region.

On physical examination, a painless mass was palpated in the abdomen, mainly in the central part of the abdomen, with non‐symmetrical distention and was dull on percussion. There was no guarding or tenderness, and evaluation of hepatomegaly or splenomegaly was not possible. The bowel sound was hypoactive and the rectal examination was empty and normal. Other following physical examinations were unremarkable. Laboratory evaluation including complete blood count and blood chemistry tests were normal. X‐ray of the chest and abdomen showed normal lungs, a large dilated distal part of the bowel without air fluid level, which was causing distal obstruction, and the liver was not seen in its anatomical region (Figure 1).

**FIGURE 2:**
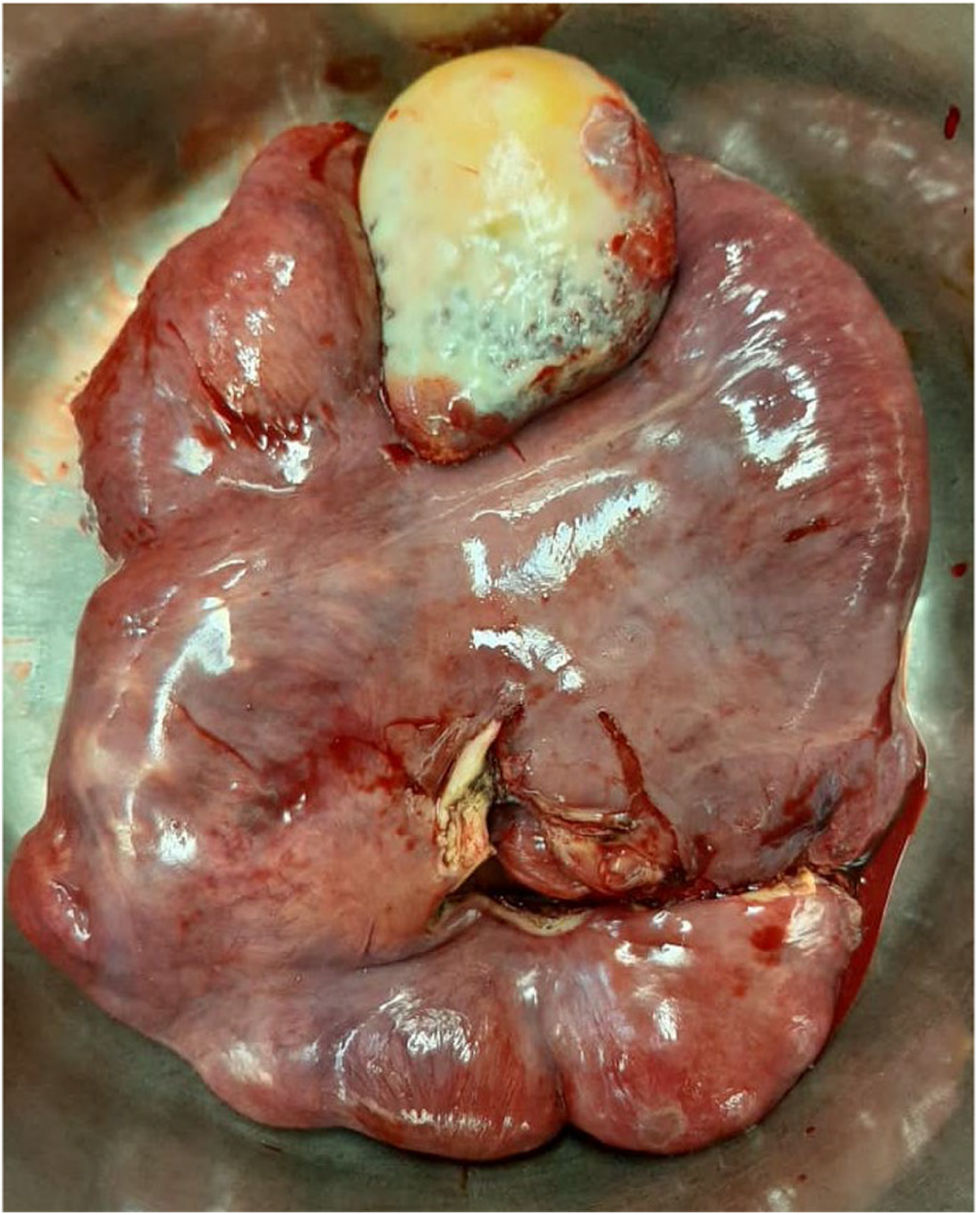
A white lesion like hydatid cyst on spleen, encapsulation of distal part of bowel and spleen cover by a dense whitish membrane.

The patient underwent exploratory laparotomy and had 130 cm of the Treitz ligament to 20 cm of the terminal ileum mass‐like lesion that contained bowel content (Figure 2). Another intraoperative finding showed a white lesion on the spleen, encapsulation of the distal part of the bowel and spleen by a dense whitish membrane, dilated transverse colon and complete rotation in the liver. The mass (encapsulated small bowel) was completely resected and enteroenteric end‐to‐end small bowel jejunoileal anastomosis was performed. The spleen was also resected due to its abnormal appearance. The liver was returned to its anatomical location, and its capsule was sutured to the parietal peritoneum with 2.0 vicryl.

**FIGURE 3:**
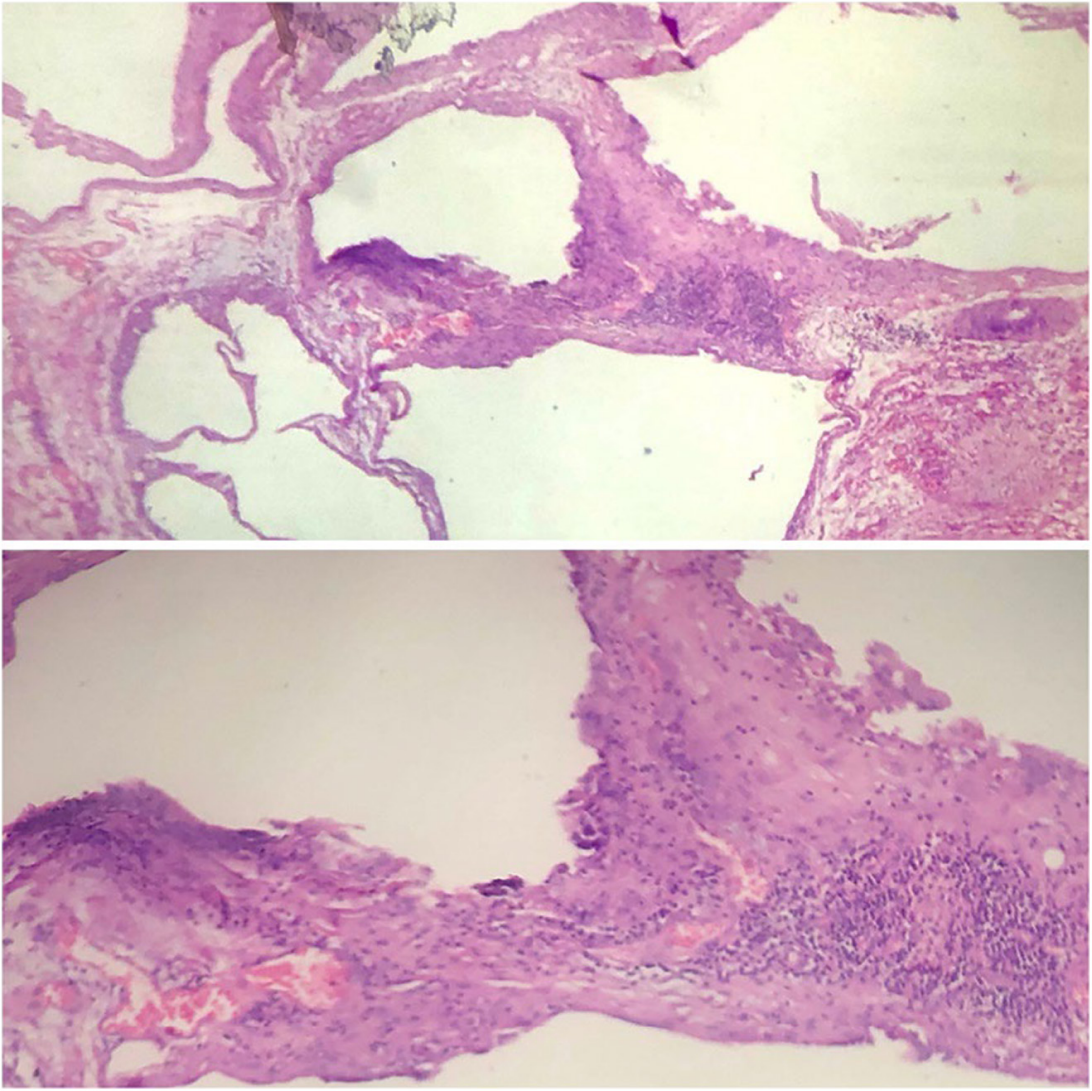
Histopathological evaluation of samples from the small intestinal, showing multiple cystic spaces lined by some histiocytic giant cells (H&E, ×200 (top) and ×400 (bottom)).

The pathology of the segmental resection membrane of the small bowel (63 cm of small intestine) showed pneumatosis cystoides intestinalis, and the pathology found in the spleen after splenectomy was perisplenitis (Figure 3). In histological evaluation, the external surface of the small bowel was gangrenous and edematous. No perforation site was seen on opening the lesion, and in some parts of the bowel we found a lesion that had a vesicular pattern with gray color. The external surface of the spleen showed white fibrin deposition and ulceration. The cytology report of the smear of the abdominal fluid was yellowish and showed a few isolated and grouped mesothelial cells, some lymphocytes, histiocytes and polymorphonuclear leukocytes, and no malignant cells were seen.

The patient's normal bowel movement and passing of flatus was restored on the third postoperative day. His nasogastric tube was successfully removed and antibiotics were halted. He was discharged on the fifth postoperative day after tolerating oral diets without any nausea or vomiting. On his 1‐year follow‐up, he reported no episodes of pain or similar attacks, gained 15 kg (more than 30%) weight, and was living a normal life.

## 3 DISCUSSION

ACS is a rare entity in which the intra‐abdominal organs become wrapped by a fibro‐collagen membrane and small bowel is the most common involved organ, the involvement of which can cause bowel obstruction.^2^ It can be classified in forms of idiopathic and secondary. The definite etiology of idiopathic ACS has not been proven; yet there are some theories suggesting the role of congenital anomalies including the absence or dysplasia of the greater omentum.^4^ Furthermore, secondary ACS is associated with irritation or inflammation of the peritoneum, characterized by acquired causes including abdominal surgery, peritoneal dialysis, infection, use of certain medications, liver cirrhosis, malignancy, gynecological diseases, organ transplantation, and autoimmune diseases.^6^ ACS can also be subdivided in three categories regarding the extent of coverage by the fibrous collagenous membrane: Type 1, the small intestine is partially covered; Type 2, the small intestine is entirely covered; and Type 3, entire small intestine and other organs are covered.^10^ In cases of severe intestinal obstruction, surgical approach remains the gold standard option, and bowel resection is indicated when necrosis has developed to prevent the fatal sequela of these conditions.^11^ In our patient's case, there were no related drug use or medical history such as beta blocker use, peritoneal dialysis, autoimmune disease, unusual infection, or surgery. Moreover, he presented with abdominal pain and protrusion, nausea, and vomiting which are classic symptoms of bowel obstruction, in addition to the abdominal X‐ray which was suggestive of severe bowel obstruction, while the diagnosis of ACS was only made during the laparotomy, and the gangrenous intestine inside the cocoon warranted the indication for resection.^6^, ^12^ The absence of an underlying condition, known to be associated with ACS, and the surgical findings suggest the diagnosis of type 1 primary ACS in our patient.

The association of ACS with intestinal malrotation is reported only once in the literature, where there is no theory to explain this co‐occurrence.^13^ Moreover, Chilaiditi syndrome is a rare cause of bowel obstruction and can be caused by either chronic constipation or congenital viscera malrotation; however, there is no association between ACS and Chilaiditi Syndrome reported in the literature.^14^ Furthermore, a fibrosed spleen which could be the result of the minor thalassemia and started the encapsulation, or could be caused by the ACS is not mentioned in the literature in association with ACS, which poses a causality dilemma that needs more investigation.

Chorti et al. conducted a systematic review of 240 reported cases of ACS and showed that the most common symptoms include diffuse abdominal pain and bowel obstruction symptomatology.^3^ Furthermore, contrast‐enhanced abdominal CT scan was significantly correlated with preoperative diagnosis of ACS which was performed in 43% of patients, and surgical management was the most common treatment for ACS cases. In another case report by Pintar et al., a case of ACS was presented with chronic abdominal pain and his abdominal CT scan was suggestive for mesenteric hernia; however, the intraoperative diagnosis was changed to ACS.^15^ Considering the acute onset and severe condition of our patient, we proceeded with the surgical approach without abdominal CT scan and reached a favorable outcome including the resolution of all symptoms and more than 30% weight gain in long‐term follow‐up.

## 4 CONCLUSION

ACS is the idiopathic encapsulation of large or small intestine and a rare cause of bowel obstruction that can accelerate the progression of bowel obstruction sequela, therefore requires early diagnosis, that can be reached by contrast‐enhanced abdominal CT scan preoperatively. The association between ACS and other congenital abnormalities including Chilaiditi syndrome requires more investigation.

## AUTHOR CONTRIBUTIONS

**Ali Tajaddini:** Data curation; investigation; project administration. **Mohammadmehdi Fallahi:** Writing – original draft; writing – review and editing. **Hoda Haghshenas:** Visualization; writing – original draft; writing – review and editing. **Soheila‐sadat Nourmohammadi:** Writing – original draft; writing – review and editing. **Leila Ghahramani:** Conceptualization; resources; supervision. **Reza Shahriarirad:** Writing – original draft; writing – review and editing.

## FUNDING INFORMATION

None.

## CONFLICT OF INTEREST STATEMENT

None to declare.

## ETHICS STATEMENT

The present study was approved by the medical ethics committee of the Shiraz University of Medical Sciences. Written informed consent was obtained from the patient regarding reporting of their data and images.

## CONSENT

Written informed consent was obtained from the patient to publish this report in accordance with the journal's patient consent policy.

## Data Availability

All data regarding the case have been reported in the manuscript. Kindly contact the corresponding author in case of requiring any further information.
